# Strategic investment patterns of the medical industry in senior health

**DOI:** 10.3389/fpubh.2025.1629981

**Published:** 2025-07-11

**Authors:** Xinyu Li, Ping Tong, Bangsen Zhu

**Affiliations:** ^1^Department of Global Trade and Management, ShinHan University, Uijeongbu-si, Republic of Korea; ^2^Xianjiang Honors School of Arts and Physical Education, Ningbo Childhood Education College, Ningbo, China; ^3^Physical Education College, ShinHan University, Uijeongbu-si, Republic of Korea

**Keywords:** senior health, strategic investment, fuzzy-set qualitative comparative analysis, medical-care integration, supply-demand coupling

## Abstract

This study constructs a fuzzy-set Qualitative Comparative Analysis (fsQCA) framework based on seven supply–demand variables in the senior-health sector of Japan and South Korea (2014–2023) and the medical-industry’s investment ratio in senior health, to systematically explore the multiple configurational pathways that enhance investment ratios. Four typical pathways were identified: P1 (high Government Fiscal Investment + Enterprise Technology Innovation Index + Older Adult Service Institution Capacity), P2 (Government Fiscal Investment + Social Capital Participation + high Health Insurance Reimbursement Rate + Older Adult Service Institution Capacity), P3 (Government Fiscal Investment + Enterprise Technology Innovation Index + Smart Service Platform Construction) and P4 (Social Capital Participation + Enterprise Technology Innovation Index + Smart Service Platform Construction + Older Adult Service Institution Capacity + high Health Insurance Reimbursement Rate). All paths demonstrate that “fiscal leverage + market incentives + digital empowerment + institutional safeguards” in four-dimensional linkage are pivotal to improving resource-allocation efficiency. Robustness tests confirm the stability of each pathway after threshold adjustments and outlier exclusions, bolstering the external validity of the findings. A Japan–Korea comparison reveals higher coverage in government-led and market-reform pathways in Korea, whereas Japan leads in the intelligent-care and telemedicine pilot pathway. Policy recommendations emphasize context-sensitive, flexible configuration of fiscal, market, technological and institutional elements, with priority support for Smart Service Platform Construction and public–private partnership models to meet differentiated needs under varying fiscal capacities and aging pressures. This study applies the dynamic fsQCA method to the integrated medical–older adult care domain, enriching the theoretical paradigm of strategic investment in the senior-health industry and offering transferable policy-configuration pathways for other aging societies.

## 1 Introduction

**Figure 1:**
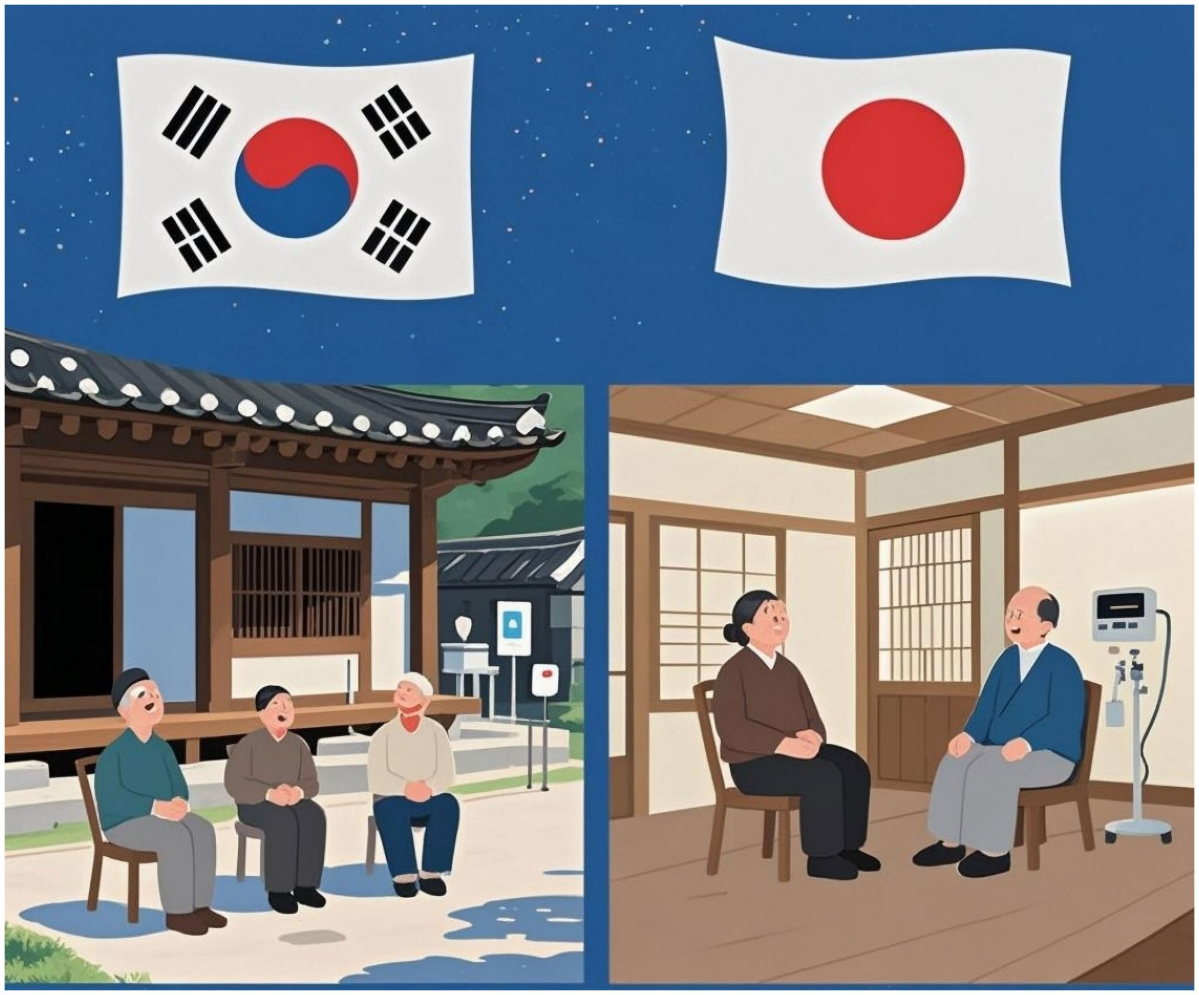
Comparison of Korea and Japan.

Strategic investment by the medical industry in the field of senior health has become a core initiative for addressing the challenges of population aging and promoting the sustainable development of public health (1). As the degree of population aging continues to rise in both South Korea and Japan, their governments and market actors have been exploring novel models of medical care integration to enhance the quality of life for older adults and optimize resource allocation (2). In South Korea, the government has introduced Social Capital Participation into older adult care services via a public private partnership model, leveraging policy subsidies, tax incentives and land grants to foster deep collaboration between medical institutions and care homes in service processes, professional training and information systems (3). Meanwhile, Japan has led the way in community based home care and the application of Smart Service Platform Construction and wearable health monitoring devices (4). Initiatives such as “Dementia Cafés” and “Community Health Stations,” together with the deployment of Internet of Things, big data and telemedicine, have established a “near at hand, accessible, continuous” service loop centered on the community (5). Although the two countries differ in institutional design and practical pathways, both demonstrate that the integration of medical industry and older adult care services not only meets increasingly diversified health needs but also drives coordinated development across the upstream and downstream of related industrial chains (6). As shown in Figure 1, this study provides an in-depth analysis of the core factors influencing the effectiveness of strategic investment. By drawing on the practical experiences of South Korea and Japan under different governance models, it offers valuable insights and practical guidance for other Asian countries entering the aging phase to develop healthcare systems tailored to their national circumstances (7).

Strategic investment in the healthcare industry for older adult well-being is directly linked to the enhancement of care quality and the long-term sustainability of public health expenditure. Against this backdrop, factors such as the degree of population aging, government fiscal input, medical insurance reimbursement rates, the bed capacity of care institutions, and the deployment of smart service platforms not only determine the scale of market demand and the intensity of policy support, but also significantly influence capital allocation and project returns (8). A systematic examination of the interplay between these critical variables and investment decisions can help clarify the underlying logic of resource distribution, providing a sound basis for optimizing and scaling integrated medical and older adult care models (9). Existing studies predominantly focus on the impact of one or two variables on strategic investment intentions in integrated older adult care, relying on qualitative approaches such as surveys and in-depth interviews, or employing quantitative techniques such as multiple linear regression, Analytic Hierarchy Process (AHP), and gray relational analysis. While a limited number of scholars have attempted to examine the issue from both the supply side of healthcare and the demand side of older adult care, most remain confined to descriptive case studies or static models, lacking the capacity to reveal the synergistic effects and path dependencies among variables (10). Moreover, key latent factors such as the degree of social capital participation and the diffusion of technological innovation remain underexplored. In response to these research gaps, this study proposes a framework encompassing seven dimensions—population aging, government fiscal input, smart service platform development, insurance reimbursement rates, institutional capacity, social capital participation, and technological innovation diffusion (11). It adopts the Qualitative Comparative Analysis (QCA) method to systematically address the core research question: “Which combinations of factors are most effective in driving higher strategic investment performance?” Specifically, the study selects representative cases of integrated older adult care initiatives in South Korea and Japan, assigning fuzzy set values to the seven variables based on official statistics, policy documents, and field interviews (12). It then constructs a truth table and applies Boolean algebra to identify core and peripheral conditions (13). Through pathway analysis and robustness testing, the study distills a series of actionable and context-sensitive configurations of influencing factors. Compared to traditional static models, QCA retains the depth of qualitative insights while enabling a rigorous examination of interactive effects, substitution mechanisms, and complementary dynamics within complex socio-economic systems (14). As such, it provides clear, evidence-based investment configurations that are highly relevant to strategic decision-making across diverse governance contexts.

The theoretical contributions of this study are twofold. First, by constructing a multidimensional impact framework encompassing population aging, policy resources, market mechanisms and technological innovation, it expands the research paradigm for strategic investment in the medical industry and senior health. Second, it is the first to apply Qualitative Comparative Analysis to this field, overcoming the limitations of single model approaches, providing a novel tool for uncovering factor synergies and path dependence, and offering a transnational comparative perspective for medical care integration practices under different governance models. Methodologically, the study’s case selection, condition calibration and truth table construction are grounded in empirical research, ensuring the credibility and operability of the findings; robustness checks via specialized software further enhance the scientific rigor and generalizability of the results. This paper fills a gap in the literature on the multi factor linkage perspective of strategic investment in the medical industry and provides theoretical support and practical guidance for governmental decision makers and private investors in resources allocation, institutional design and technology application.

## 2 Theoretical background and applied practice of investment by the medical industry in the field of senior health

### 2.1 Research background and theoretical foundations

As global demographic structures continue to age, strategic investment in the healthcare industry’s older adult care segment has emerged as a pivotal means of alleviating public health burdens and enhancing the quality of eldercare services (15). Conventional studies have predominantly concentrated on the impact of individual variables upon investment intentions, yet they have overlooked the joint mechanisms by which institutional environments and market demand interact, rendering them unable to explain why regions with comparable policy incentives can nonetheless exhibit markedly divergent investment outcomes (16). To address this lacuna, the present study adopts a “supply–demand coupling” perspective, endeavoring to transcend single-factor frameworks by integrating institutional incentives with market pressures, thereby revealing the driving mechanisms of integrated medical–older adult care investment (17). However, absent a robust theoretical underpinning, such coupling analyses risk appearing arbitrary and lack generalizability. Accordingly, this section draws upon the dual theoretical lenses of New Institutional Economics and the Resource–Capability View to afford the coupling model a firm scholarly foundation, and to demonstrate why incorporating the seven variables—government fiscal investment, social capital participation, technological innovation, smart platform deployment, institutional capacity, insurance reimbursement rates, and population aging—into a unified analytical framework is logically coherent (18).

From the standpoint of New Institutional Economics (NIE), institutional settings decisively influence resource allocation and transaction costs (19). Government fiscal investment signifies official support for the older adult healthcare sector, encompassing budget allocations, tax relief and land grants, thus directly reducing project transaction costs; social capital participation reflects non-governmental entities’ risk preferences and resource-integration capabilities in public service projects, with its scale and structure exerting a profound impact on financing pathways and project feasibility; and the enterprise technology innovation index, as a manifestation of firm competitiveness within prevailing institutional frameworks, not only shapes the development and dissemination of medical devices and smart eldercare solutions but also determines service efficiency and quality control (20). Consequently, treating government fiscal investment, social capital participation and technological innovation as supply-side variables allows for a comprehensive grasp of how institutional incentives mold the sector’s supply capacity (21). Meanwhile, under the Resource–Capability View, strategic decision-making hinges upon the alignment of an organization’s internal resource endowments with external market demands. On the demand side, smart service platform construction showcases the extent to which digital technologies—such as teleconsultation, big-data health analytics and interoperable electronic health records—reengineer care delivery processes, reducing coordination costs and enhancing service accessibility; older adult service institution capacity gages the physical resource base of the market through indicators such as available beds and day-care center numbers, directly correlating to service scale and marginal returns; health insurance reimbursement rates, as a core payment-protection metric, determine elders’ out-of-pocket costs and willingness to utilize services; and the degree of population aging, measured by the proportion of those aged 65 and over, reflects potential demand scale and market expansion (22). By regarding these four metrics as demand-side variables, one can accurately capture market pressures driving strategic investment and reveal the dynamic matching between supply incentives and demand forces.

Within our analytical framework, the supply- and demand-side variables collectively constitute the multidimensional drivers of integrated care investment. The supply side comprises three key elements—government fiscal investment, social capital participation and the technology innovation index—each quantifiable by budgetary outlays, PPP project counts, private capital inflows, R&D intensity, patent filings and technology adoption rates (23). The demand side involves four factors—smart platform coverage (measured via user numbers or module counts), institutional capacity (beds and facility counts), insurance reimbursement ratios and population aging degree—each capturing distinct aspects of market need and service uptake (24). These seven variables, when modeled together, not only elucidate how institutional incentives influence funding and technological progress drives service evolution, but also how digital platforms, facility resources and payment assurances shape demand, with population aging providing long-term trend forecasts, thereby enabling a systematic supply–demand coupling analysis of investment performance (25).

Analysing these seven variables within a single model stems from the complementary nature of the two theoretical perspectives and the inherent systemic attributes of integrated care. NIE emphasizes the efficacy of institutional constraints and incentive designs, while the Resource–Capability View focuses on resource utilization and capability transformation (26). In the healthcare–eldercare nexus, government and social capital furnish institutional and financial backing, technological innovation and smart platforms spur service innovation, institutional capacity and reimbursement rates jointly determine market absorptive capacity, and population aging offers the longitudinal context. Incorporating all seven variables into a unified Qualitative Comparative Analysis framework allows us to discern their relative importance across diverse institutional and market settings, to distinguish their combinatory and substitution effects, and to furnish policymakers and investors with practical configuration pathways—ultimately providing both theoretical and empirical support for strategic investment in integrated older adult healthcare across Asia and beyond.

### 2.2 Investment practice by the medical industry in the field of senior health

With the intensification of population aging, investment practice by the medical industry in senior health in Japan has undergone a profound transformation from single-product supply to the provision of comprehensive solutions (27). Japanese corporations such as Panasonic Healthcare and Fujitsu have not only increased their stakes in high-end medical diagnostic devices and wearable health-monitoring terminals but have also formed strategic alliances with municipal retirement communities and hospital networks to construct data-driven, end to end health management service systems. Panasonic Healthcare, leveraging its expertise in sensor manufacturing and home appliance miniaturization, has launched a smart wristband capable of real time monitoring of blood pressure, blood glucose and activity levels (28). In collaboration with local governments, it is piloting a dual track “home + remote” care model for older adult patients with severe chronic illnesses in over 20 communities, thereby forming an integrated “device platform service” loop (29). Fujitsu, on the other hand, has capitalized on its strengths in cloud computing and artificial intelligence to partner with leading Japanese universities and multiple hospitals in the development of a “Smart Senior Health Platform.” By applying large scale machine learning to vital, behavioral and environmental data from tens of thousands of older users, it has developed early warning models that predict fall risk and acute heart failure episodes (30). In conjunction with insurance providers, these models have been embedded into chronic disease management insurance products, delivering precise interventions for high risk groups. These initiatives have markedly reduced emergency visits and readmission rates at community hospitals while providing service based, recurring revenue streams unequivocally demonstrating the decisive role of deep technological integration and multi stakeholder collaboration in creating value within the senior health industry (31).

In South Korea, leading medical industry firms have similarly integrated themselves deeply into senior health service systems through cross sector mergers and acquisitions, equity investments and platform co development, rapidly enhancing their competitiveness in the older adult care market. Samsung Life, for instance, utilizes its capital and risk management capabilities in financial services to invest heavily in domestic Medical Care Integration operators, taking part in the establishment of multiple medical rehabilitation long term care communities (32). By underwriting a portion of operational risk with proprietary insurance products, Samsung Life has created a dual revenue model of “service income + insurance premium income.” For data interoperability, it has connected its investments to its financial risk control systems, enabling real time health monitoring and dynamic risk assessment of residents, so that insurance payouts and health interventions are synchronously triggered substantially lowering long term care premium costs (33).

LG CNS, renowned for its IT and system integration prowess, has teamed with a national university hospital and regional older adult care centers to develop an “Intelligent Senior Care Cloud Platform.” This platform integrates electronic medical records, cloud based medical imaging, virtual consultation suites and social rehabilitation features for older adults. It not only achieves a full process data loop but also provides standardized interfaces for third party service providers such as domestic help, meal subscription services and psychological counseling thereby accelerating ecosystem expansion. By the end of 2024, this platform had reached over 50 municipal districts, serving more than 100,000 senior users and generating stable cloud service subscription revenues for LG CNS (34).

Although Japanese and South Korean enterprises emphasize different facets, their investment practice in senior health collectively reflects three strategic pillars: “Technology Empowerment + Ecosystem Collaboration + Risk Sharing.” In terms of Technology Empowerment, firms in both countries have upgraded single-point medical devices into sustainable health management terminals via IoT, artificial intelligence and big data analytics, consolidating disparate data onto unified platforms to deliver personalized, preventive and continuous interventions (35). In Ecosystem Collaboration, partnerships such as Panasonic Healthcare with municipal retirement communities and Samsung Life with Medical Care Integration operators demonstrate that companies are no longer operating in isolation but are building symbiotic networks across the value chain and industries to share costs and jointly reap benefits (36). In Risk Sharing, by organically combining insurance capital, governmental subsidies and user fees, enterprises can distribute operational risk while guaranteeing service quality, and offer predictable returns to both public and private investors thereby fostering a virtuous cycle driven by policy incentives and market orientation (37). From a supply demand theoretical perspective, these successful practices not only balance strong demand among older adults for high quality health services with their payment capacity but also enhance overall supply efficiency through the organic allocation of public and private resources. They provide replicable experience models for innovation in the Medical Care Integration industrial paradigm (38).

## 3 Methodology and analysis

### 3.1 Data sample framework construction

**Figure 2:**
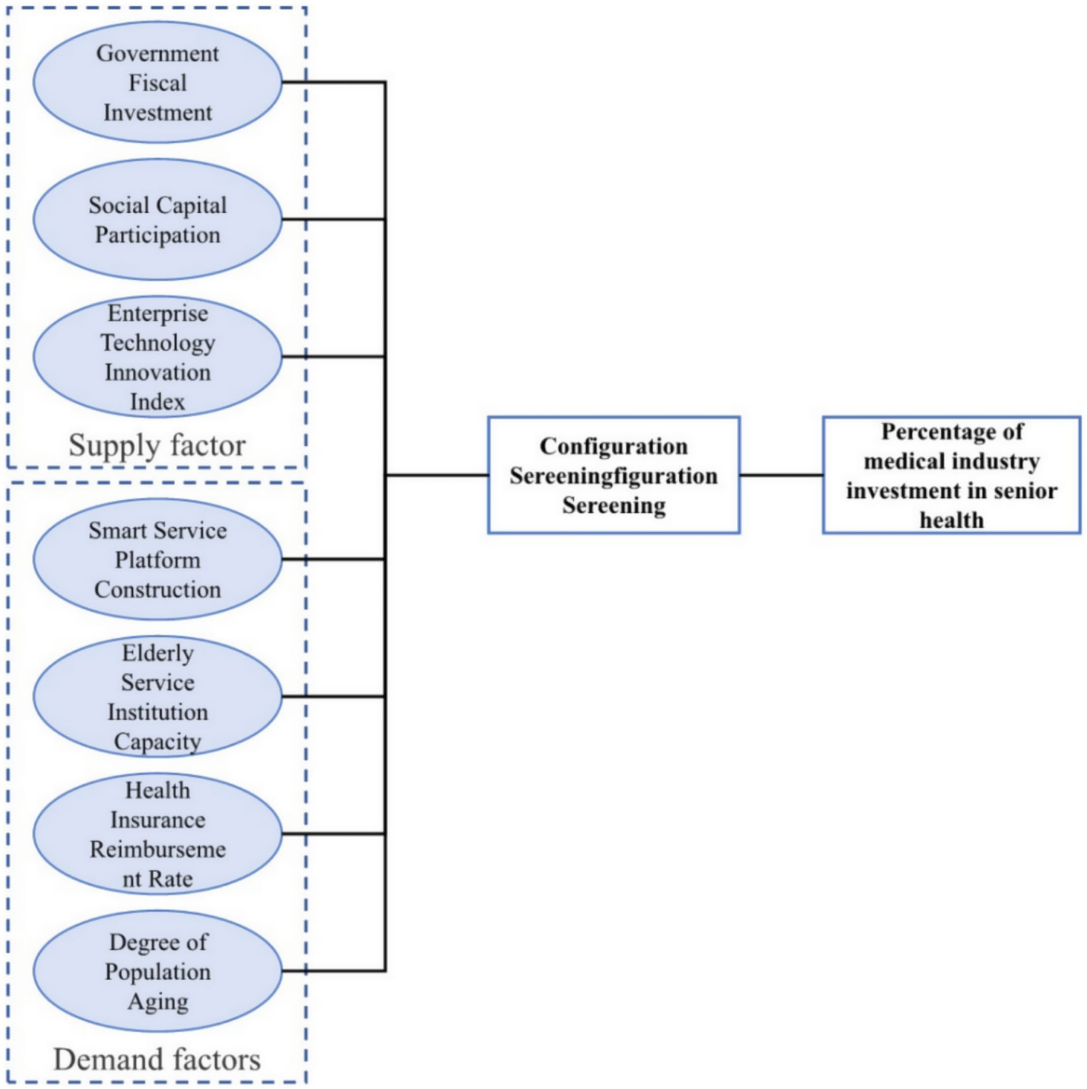
Data framework construction diagram.

With the acceleration of population aging, senior health has become a central concern for both governments and market actors worldwide. High income nations particularly Asian exemplars South Korea and Japan, each endowed with mature public health systems and information based regulatory mechanisms began systematic strategic investment in the senior health sector as early as 2014 (39). As shown in Figure 2, This study constructs its data sample framework from a dual supply demand perspective, selecting three supply side factors Government Fiscal Investment (GFI), Social Capital Participation (SCP) and Enterprise Technology Innovation Index (ETII) and four demand side factors Smart Service Platform Construction (SSPC), Older Adult Service Institution Capacity (ESIC), Health Insurance Reimbursement Rate (HIRR) and Degree of Population Aging (DPA). We compile annual panel data for both countries over the period 2014 2023 to undertake a horizontal, country level comparison (40, 41).

Firstly, South Korea leverages the multidimensional data resources of the National Health Insurance Service (NHIS) and the Health Insurance Review & Assessment Service (HIRA) to monitor in real time each year’s Government Fiscal Investment, service utilization and reimbursement patterns. Japan, in turn, relies on the Ministry of Health, Labor and Welfare and the mandatory annual surveys under the Long Term Care Insurance (LTCI) system to assemble a comprehensive database encompassing fiscal allocations, social donations, institutional capacity and digital platform metrics (42). Secondly, to ensure national representativeness and comparability of indicators, all data sources are verified against official statistical releases from each country and OECD databases (43). This guarantees that flow based metrics such as GFI and SCP, technological and platform development indices such as ETII and SSPC, service capacity and payment security indicators such as ESIC and HIRR, and demographic structure measures such as DPA, are all drawn from transparent, traceable official documents and international reports (44). On this basis, comparative analysis of the two countries’ funding intensity trajectories, market participation rates and innovation vigor enables a deep dissection of the interaction mechanisms among government policy orientation, private sector incentives and industrial technological advancement (45). It also permits exploration of the co evolutionary pathways of senior health service supply and demand under different institutional frameworks, thereby offering transferable lessons and policy recommendations to other nations confronting rapid population aging (46).

For the seven core indicators, rigorous quantitative methods and standardization procedures are applied. Government Fiscal Investment (GFI) is measured by each country’s annual “Health and Aging Services Special Budget” expenditure, with nominal values converted to current year US dollars via purchasing power parity (PPP) to neutralize the effects of inflation and exchange rate fluctuations. Year on year growth rates of these expenditures are then calculated to reveal the temporal dynamics of policy support intensity. Social Capital Participation (SCP) is defined as the proportion of each special budget sourced from private capital, charitable foundations and venture funds, directly reflecting market actors’ willingness to enter and the depth of their engagement; numerator and denominator data for SCP are obtained from annual reports published by finance ministries, relevant foundations and industry associations (47). The Enterprise Technology Innovation Index (ETII) integrates R\&D expenditure ratios and targeted R\&D investments in smart medical devices, rehabilitation robotics and digital diagnostics. On the demand side, Smart Service Platform Construction (SSPC) is indexed by the installed base of IoT enabled monitoring devices, the number of AI driven health management platforms serving hospitals and communities, and the proportion of telemedicine systems integrated into medical consortiums; these components are standardized and aggregated. Older adult Service Institution Capacity (ESIC) is expressed as the number of beds or service slots per 1,000 individuals aged 65 and over, indicating physical service capacity. Health Insurance Reimbursement Rate (HIRR) takes each year’s average reimbursement percentage of senior citizen medical expenditure under national health insurance, representing institutional payment security. Degree of Population Aging (DPA) is the share of the population aged 65 + in the total population (48).

To mitigate confounding macro environmental influences, this study employs a dynamic QCA (Qualitative Comparative Analysis) approach on the constructed panel data framework, systematically analysing the interactive effects of supply side conditions (GFI, SCP, ETII), demand side provisions (SSPC, ESIC, HIRR) and demographic pressure (DPA). This facilitates an investigation of each nation’s systematic investment strategy in senior health at the country level (49).

### 3.2 Data calibration

As shown in Table 1, In dynamic fuzzy-set qualitative comparative analysis (fsQCA), direct calibration is pivotal for mapping raw variables onto the [0, 1] membership-degree scale in a manner that is both rigorous and robust, thereby accommodating sample heterogeneity and ensuring model comparability. Accordingly, this study adheres to established methodological practice by designating each variable’s 95th, 50th, and 5th percentiles as its “full membership,” “crossover point” and “full non-membership” anchors (50). On the one hand, the 95th and 5th percentiles effectively eschew distortions from extreme maxima and minima, enhancing calibration stability; on the other hand, the 50th percentile serves as the fuzzy-set midpoint—neither fully in nor fully out—ensuring a smooth transition in the membership function that faithfully reflects typical regional and variable-level distinctions across both supply and demand dimensions. Employing the anchor criteria proposed by Liu and Yi (51), we directly calibrated seven condition variables—government fiscal investment (A), social capital participation (B), enterprise technology innovation index (C), smart service platform construction (D), older adult service institution capacity (E), health insurance reimbursement rate (F) and degree of population aging (G)—together with the outcome variable, the proportion of medical-industry investment in older adult healthcare (Y). The procedure entailed first extracting the 95th/50th/5th percentile values from each variable’s raw data as the critical thresholds for the membership function; subsequently, we applied a standard fuzzy-set transformation to map these thresholds into the [0, 1] interval, thereby ensuring comparability across differing units and data sources.

**Table 1:** Calibration of variables.

Variables	Variable name	Fully affiliated	Intersection	Completely unaffiliated
Outcome variables	The medical industry’s investment ratio in senior health	0.135	0.043	0.014
Condition variable	Government fiscal investment	0.143	0.024	0.017
	Social capital participation	0.437	0.153	0.253
	Enterprise technology innovation index	0.427	0.156	0.273
	Smart service platform construction	1214.134	512.18	183.960
	Older adult service institution capacity	2143.221	137.135	121.793
	Health insurance reimbursement rate	0.713	0.485	0.264
	Degree of population aging	14.682	11.231	8.256

Calibration results are as follows: Supply-side: A’s three anchors are 0.143/0.024/0.017, indicating a generally low and tightly clustered distribution; B’s are 0.437/0.153/0.253, showing high discriminative power; C’s are 0.427/0.156/0.273—similar to B—revealing pronounced regional variation in market-driven and innovation-driven factors: Demand-side: D’s anchors (1214.134/512.180/183.960) and E’s (2143.221/137.135/121.793) exhibit vast spans, reflecting considerable regional divergence in technological platforms and service-supply infrastructure; F’s (0.713/0.485/0.264) reveal stratified policy-protection intensity; G’s (14.682/11.231/8.256) capture differences in demographic pressure. Outcome variable: Y’s anchors are 0.135/0.043/0.014. Although overall low, Y possesses sufficient variance for use in subsequent causal-path analysis.

This calibration not only underscores the background-setting and reinforcing roles of the supply-side core elements (A, B, C), but also illustrates how demand-side factors (D, E, F, G) jointly shape investment behavior via technological platforms, institutional capacity, policy-based protection and demographic pressure. Building on this calibrated foundation, the forthcoming dynamic fsQCA will investigate multiple causal pathways to furnish robust empirical support for precision policy design and resource allocation (52).

### 3.3 Necessity analysis of the data sample

Within the panel data framework of Qualitative Comparative Analysis (QCA), necessity analysis seeks to ascertain whether a given condition (or its absence) is consistently present across all cases exhibiting the target outcome. Its principal metric is consistency, for which a threshold of 0.90 is typically adopted to designate a necessary condition; coverage then gages the substantive explanatory power of that condition for the outcome (53). As shown in Table 2, According to configurational theory grounded in Boolean algebra and set theory, reducing calibration distance can enhance the precision of consistency estimates, thereby more accurately identifying necessary conditions. Should a condition meet the consistency threshold with moderate coverage, it may be provisionally deemed a necessary precursor to the outcome.

**Table 2:** Data necessity test.

Variant	Aggregate consistency	Aggregate coverage	Inter-group consistency	Intra-group consistency
The medical industry’s investment ratio in senior health (Y)				
A	0.65	0.71	0.05	0.05
~A	0.84	0.62	0.02	0.04
B	0.75	0.60	0.03	0.03
~B	0.67	0.62	0.04	0.05
C	0.74	0.73	0.03	0.06
~C	0.72	0.68	0.02	0.07
D	0.74	0.67	0.04	0.06
~D	0.69	0.62	0.05	0.07
E	0.73	0.62	0.03	0.04
~E	0.84	0.67	0.04	0.04
F	0.82	0.62	0.03	0.03
~F	0.81	0.63	0.05	0.04
G	0.76	0.68	0.04	0.03
~G	0.68	0.61	0.03	0.04
The medical industry’s investment ratio in senior health (~Y)				
A	0.78	0.43	0.01	0.05
~A	0.72	0.66	0.03	0.03
B	0.70	0.35	0.02	0.06
~B	0.80	0.62	0.04	0.04
C	0.72	0.62	0.02	0.02
~C	0.75	0.64	0.03	0.05
D	0.73	0.61	0.04	0.06
~D	0.77	0.64	0.06	0.07
E	0.64	0.62	0.02	0.05
~E	0.78	0.60	0.07	0.04
F	0.82	0.53	0.05	0.07
~F	0.71	0.65	0.04	0.06
G	0.72	0.61	0.07	0.06
~G	0.65	0.71	0.05	0.04

For the necessity test of “high medical industry investment ratio in senior health (Y),” no single condition or negation thereof achieves a consistency above 0.90. The highest consistencies are observed for “\ ~ A (low Government Fiscal Investment)” and “\ ~ E (low Older Adult Service Institution Capacity),” each at 0.84 with coverages of 0.62 and 0.67 respectively; “F (high Health Insurance Reimbursement Rate)” records a consistency of 0.82 and a coverage of 0.62. Although these conditions frequently appear in high Y cases, none satisfies the threshold for necessity, indicating that a high investment ratio is not driven by any solitary factor but rather by the synergistic action of multiple conditions (54).

Similarly, for the necessity test of “low medical industry investment ratio in senior health (\ ~ Y),” no condition or its negation exceeds a consistency of 0.90. The negations “\ ~ B (low Social Capital Participation)” and “\ ~ D (low Smart Service Platform Construction)” exhibit consistencies of 0.80 and 0.77 with coverages of 0.62 and 0.64 respectively; other indicators display consistencies predominantly in the 0.65 0.75 range. Thus, low investment ratios are likewise not determined by any single necessary condition. Overall, the sample warrants close attention to those conditions with relatively high consistency (>0.80) yet falling short of the threshold, as these will inform the empirical basis for multi condition configurational testing in the subsequent fsQCA pathway analysis (55).

### 3.4 Configurational analysis

As shown in Table 3, prototypical high-income nations in Asia, South Korea and Japan, leveraging their robust public-health systems and digital governance mechanisms, initiated systematic strategic investment in the older adult-healthcare sector as early as 2014 under their respective National Health Insurance Service (NHIS/HIRA) and Long-Term Care Insurance (LTCI) schemes. This study adopts a dual supply–demand perspective, selecting three supply-side variables—Government Fiscal Investment (GFI), Social Capital Participation (SCP) and Enterprise Technology Innovation Index (ETII)—and four demand-side variables—Smart Service Platform Construction (SSPC), Older Adult Service Institution Capacity (ESIC), Health Insurance Reimbursement Rate (HIRR) and Degree of Population Aging (DPA)—to construct an annual panel dataset for both countries covering 2014–2023. Employing dynamic fuzzy-set qualitative comparative analysis (fsQCA), we distill four prototypical configurational pathways (Parameterizations 1–4) and quantitatively evaluate each pathway’s consistency, original coverage, PRI score, and adjusted within- and between-case distances, thereby elucidating diverse combination patterns for achieving a high proportion of medical-industry investment in older adult healthcare under varying policy imperatives and demographic pressures.

**Table 3:** Configuration analysis results.

Conditional variables	Parameterization 1	Parameterization 2	Parameterization 3	Parameterization 4
(A) Government fiscal investment	●	●	●	●
(B) Social capital participation		●	⊗	●
(C) Enterprise technology innovation index	●		●	●
(D) Smart service platform construction	⊗	●	⊗	
(E) Older adult service institution capacity	●		●	●
(F) Health insurance reimbursement rate		●		
(G) Degree of population aging	●	●	●	●
Consistency	0.850	0.821	0.912	0.815
Original coverage	0.302	0.339	0.219	0.264
Unique coverage	0.056	0.127	0.032	0.032
PRI	0.624	0.577	0.631	0.641
Inter-group consistency adjusted distance	0.052	0.024	0.021	0.017
Intra-group consistency-adjusted distance	0.034	0.025	0.037	0.024
Overall PRI	0.613			
Overall consistency	0.832			

Across the four prototypical configurational pathways identified in this study (P1–P4), each exemplifies the interactive mechanisms and policy implications of supply- and demand-side factors under varying developmental stages and institutional contexts. Firstly, Pathway 1 (P1) is driven primarily by high Government Fiscal Investment (GFI), high Enterprise Technology Innovation Index (ETII) and high Older Adult Service Institution Capacity (ESIC); despite low Social Capital Participation (SCP), Smart Service Platform Construction (SSPC) and Health Insurance Reimbursement Rate (HIRR), a pronounced Degree of Population Aging (DPA) still delivers a high investment ratio (Consistency = 0.850, Coverage = 0.302). This configuration typifies the early reform phase, wherein the government leads by deploying special budgetary measures to support technology firms and rapidly expanding offline beds and care slots to fill the gaps left by nascent private capital and digital services. Secondly, Pathway 2 (P2) combines high GFI with high SCP alongside elevated ESIC and HIRR; even with ETII and SSPC at nascent levels, the “capital + institution” dual-engine model maximizes investment efficiency (Consistency = 0.821, Coverage = 0.339). This configuration illustrates the ‘amplifier’ effect of robust institutional safeguards on private capital participation—underpinned by mature public insurance and regulatory frameworks in South Korea and Japan—whereby social capital secures more reliable return expectations and, through compliant operation, drives simultaneous growth in project scale and service quality. Pathway 3 (P3) emphasizes the triad of “government-led + corporate innovation + smart platforms”: in the context of significant aging pressure, high GFI, ETII and SSPC synergise to sustain high investment ratios via intelligent care and telemedicine pilots, even when ESIC and HIRR are relatively weak (Consistency = 0.912, Coverage = 0.219). This configuration, exemplified by Japan’s community-based, Internet + healthcare models, demonstrates the transformative potential of digital technologies on traditional care paradigms. Finally, Pathway 4 (P4) highlights the substitutive and complementary roles of market mechanisms and insurance capital: with comparatively limited GFI, a coalition of high SCP, advanced technology (ETII), mature SSPC, ample ESIC and high HIRR collectively compensates for fiscal shortfalls, sustaining elevated investment ratios (Consistency = 0.815, Coverage = 0.264). This pathway indicates that, when public resources are constrained, diversified market actors and comprehensive payment-protection schemes can collaboratively construct a ‘community-oriented’ eldercare ecosystem, integrating private capital with public services. Collectively, these configurations reveal that under different policy stimuli and demographic pressures, government funding, market dynamism and technological innovation achieve dynamic equilibrium through multiple combinations, with smart platforms, institutional capacity and protection mechanisms forming the cornerstones of sustainable investment growth. For policymakers, it is advisable to adapt and refine these configurational elements in accordance with national or regional fiscal capacity, social-capital market maturity and digital infrastructure readiness to attain optimal and enduring allocations of medical-industry investment in older adult healthcare.

Comparative analysis of these four pathways yields the following policy implications: first, governments should fully leverage fiscal levers in synergy with social capital and technological innovation, avoiding “isolation” of single policy tools; second, under high aging pressure, priority support for SSPC via digital means can enhance resource utilization in medical and long term care; third, when aging pressure is lower or fiscal space is constrained, raising HIRR and ESIC alongside diversified private capital incentives can achieve “market + institution” complementarity; finally, policy packages should be tailored flexibly to different institutional frameworks and developmental stages to meet heterogeneous regional needs.

### 3.5 Configuration robustness analysis

To verify the reliability and robustness of the principal model’s configurational results, As shown in Table 4 this study conducted multiple sensitivity tests on key parameters namely the consistency threshold, calibration anchor points and case inclusion beyond the original four typical pathways (Parameterization 1 4). Post-testing, the consistency values for the four configurations remained stable between 0.773 and 0.853, closely aligning with the main model’s range of 0.815 0.912. The original coverage fluctuated only modestly between 0.211 and 0.254, versus 0.219 0.339 in the primary analysis, indicating that the breadth of each pathway’s explanatory reach was not materially reduced by parameter tweaks. PRI values also held steady at 0.475 0.547, comparable to the initial 0.577 0.641, demonstrating that the discriminative power and directional clarity of each pathway remained intact. Furthermore, the maximum adjusted between-set distance stayed at a mere 0.031, and the highest within-set distance was only 0.024 figures akin to the original model’s between 0.017 0.052 and within 0.025 0.037 ranges underscoring that the granularity and consistency characteristics of the causal configurations were preserved despite anchor repositioning and outlier exclusion.

**Table 4:** Configuration robustness analysis results.

Conditional variables	Parameterization 1	Parameterization 2	Parameterization 3	Parameterization 4
(A) Government fiscal investment	●	●	●	
(B) Social capital participation			⊗	●
(C) Enterprise technology innovation index	●	●	●	●
(D) Smart service platform construction	⊗	●	⊗	⊗
(E) Older adult service institution capacity	●		●	●
(F) Health insurance reimbursement rate		●		
(G) Degree of population aging	●	●		●
Consistency	0.817	0.792	0.853	0.773
Original coverage	0.242	0.234	0.211	0.254
Unique coverage	0.031	0.116	0.028	0.027
PRI	0.526	0.475	0.547	0.516
Inter-group consistency adjusted distance	0.031	0.016	0.020	0.015
Intra-group consistency-adjusted distance	0.021	0.019	0.024	0.021
Overall PRI	0.506			
Overall consistency	0.812			

These robustness checks confirm that all four identified pathways “Government Fiscal Investment + Enterprise Technology Innovation Index + Older Adult Service Institution Capacity,” “Government Fiscal Investment + Social Capital Participation + Health Insurance Reimbursement Rate,” “Government Fiscal Investment + Enterprise Technology Innovation Index + Smart Service Platform Construction” and “Social Capital Participation + Enterprise Technology Innovation Index + Health Insurance Reimbursement Rate + Smart Service Platform Construction” exhibit high consistency and replicability, remaining resilient to parameter adjustments and sample variations. This consistency across theoretical thresholds and data-quality controls affirms the empirical support for our causal-configurational analysis of systematic strategic investment in senior health in Japan and South Korea, and further enhances the external validity and policy relevance of our findings.

### 3.6 Country coverage analysis

Based on the 2014 2023 fsQCA analysis for senior health in South Korea and Japan, the four typical configurations (P1 P4) exhibit marked national differences in coverage. As shown in Table 4 South Korea outperforms Japan in Configurations 1, 2 and 4, with Configuration 4 (market + high end technology + digitalization + capacity + reimbursement) achieving the highest coverage of 0.478. This indicates that when Government Fiscal Investment is constrained, Korea swiftly fills public service gaps by liberalizing insurance capital and pursuing market reforms. By contrast, Japan’s coverage distribution is more balanced, with no single pathway dominating. This divergence not only reveals diversified policy combinations employed by the two countries in response to rapid aging but also underscores the crucial role of institutional design and implementation pace in raising the medical industry investment ratio in senior health.

As shown in Table 5, a comparison of configurations shows that P1 (“Government Fiscal Investment + Enterprise Technology Innovation Index + Older Adult Service Institution Capacity”) has a coverage of 0.317 for Korea versus 0.233 for Japan: Korea’s earlier use of special budgets to expand beds and care slots significantly boosted initial investment efficiency, whereas Japan relied more on subsequent intelligent retrofitting. P2 (“Government Fiscal Investment + Social Capital Participation + Health Insurance Reimbursement Rate + Older Adult Service Institution Capacity”) scores 0.421 for Korea and 0.377 for Japan: Korea’s active attraction of private capital and service expansion drove a “funding + institution” dual engine, whereas Japan’s regional reimbursement discrepancies and cautious capital admission slightly dampened private-sector incentives. P3 (“Government Fiscal Investment + Enterprise Technology Innovation Index + Smart Service Platform Construction”) is higher in Japan (0.325) than Korea (0.273): Japan’s large-scale pilots in telemonitoring and AI diagnostics since 2017 have made smart services central to addressing deep aging; Korea, although accelerating digitalization, remains limited by institutional capacity and reimbursement. The most pronounced gap appears in P4 (“Social Capital Participation + Enterprise Technology Innovation Index + Smart Service Platform Construction + Older Adult Service Institution Capacity + Health Insurance Reimbursement Rate”), at 0.478 for Korea versus 0.327 for Japan: Korea’s market reforms and insurance-capital openness have yielded a richer diversity of service provision, whereas Japan’s reform has proceeded more cautiously due to institutional inertia and decentralization.

**Table 5:** Configuration country coverage analysis.

Configuration 1	Configuration 2	Configuration 3	Configuration 4
0.317	0.421	0.273	0.478
0.233	0.377	0.325	0.327

In light of these coverage disparities, policy design should first implement differentiated support: regions experiencing deep aging may emulate Japan’s mature Smart Service Platform Construction and telecare pilots by increasing investment in AI assisted diagnosis and digital health management; areas under fiscal strain but with strong market vigor could follow Korea’s market reform path by relaxing insurance and private capital entry thresholds to leverage diverse funding for service provision. Simultaneously, coordination between Older Adult Service Institution Capacity development and Health Insurance Reimbursement Rate policies must be strengthened by modestly raising bed and care slot standards and streamlining reimbursement procedures to ensure service supply matches demand. Building a closed loop mechanism encompassing Government Fiscal Investment, Social Capital Participation, Health Insurance Reimbursement Rate and digital services is essential to drive cross-departmental regulatory collaboration. For configurations with lower coverage, emphasis should be placed on bolstering Enterprise Technology Innovation Index support and enhancing public private partnership (PPP) management capabilities. Finally, under a dynamic evaluation and localized framework, fsQCA and other mixed quantitative qualitative methods should be continuously applied to monitor each pathway’s fit at different development stages and flexibly optimize factor combinations according to local institutional foundations, demographic profiles and fiscal capacity thereby achieving the optimal balance between resource efficiency and service quality in senior health.

## 4 Discussion

This study utilizes a dynamic fuzzy-set qualitative comparative analysis (fsQCA) to systematically uncover the multifaceted synergistic mechanisms among seven dimensions: government fiscal investment, social capital participation, enterprise technology innovation index, smart service platform construction, older adult service institution capacity, health insurance reimbursement rate, and degree of population aging. Our findings demonstrate that no single factor can independently drive high levels of medical-industry investment in senior health; only the four-dimensional linkage of “fiscal leverage + market incentives + digital empowerment + institutional safeguards” can markedly enhance resource allocation efficiency and policy effectiveness. Comparative analysis between Japan and South Korea reveals that South Korea achieves greater configurational coverage in the government-led (P1), capital-institution dual-engine (P2) and multi-compensation (P4) pathways, reflecting its advantage in rapidly expanding care resources and mobilizing insurance capital via subsidies and tax incentives, yet still requires improvements in smart-platform deployment and digitalized reimbursement. Conversely, Japan excels in the “innovation + platform-led” (P3) pathway, having accrued substantial expertise in telemedicine and smart eldercare—particularly in AI-assisted diagnostics, chronic-disease management and insurance payment reform.

For South Korea, while continuing to leverage fiscal support and private investment, priority should be given to integrating smart platforms within the national insurance scheme by introducing differentiated reimbursement schedules and performance-based bonuses, thereby incentivizing remote monitoring, AI-enabled diagnostics and big-data analytics. Simultaneously, a “Government–Social Capital Elder-Care Fund” and risk-sharing mechanisms should be established to lower barriers for private investors and encourage the construction of high-standard care hubs in under-served areas, ensuring a balanced expansion of institutional capacity and digital services. For Japan, to capitalize on its smart-care and telemedicine lead, regulatory barriers to social and insurance capital entry should be eased: the national insurance system ought to broaden coverage to include smart-care devices and remote-service fees, while regulatory sandboxes could be introduced to pilot novel service models and commercial insurance products. Furthermore, Japan should adopt South Korea’s model of fiscal subsidies and tax breaks for capacity expansion, promoting public–private partnerships to augment bed capacity in response to rising care demand. Both nations should harness data interoperability and policy coordination to forge a sustainable integrated care system anchored on “fiscal leverage + market incentives + digital empowerment + institutional safeguards.”

## 5 Conclusion and implication

### 5.1 Conclusion

Through dynamic fsQCA analysis of seven supply demand variables and investment ratios in the senior health sector of South Korea and Japan (2014, 2023), this study uncovers the driving mechanisms by which combinations of Government Fiscal Investment, Social Capital Participation, Enterprise Technology Innovation Index, Smart Service Platform Construction, Older Adult Service Institution Capacity, Health Insurance Reimbursement Rate and Degree of Population Aging yield a high medical industry investment ratio in senior health.

First, configurational pathways demonstrate that single policy instruments cannot independently achieve a high investment ratio; coordinated action across the four dimensions of “fiscal leverage + market incentives + digitalization + institutional safeguards” is indispensable. P1 and P2 reveal the complementary effect of concurrent Government Fiscal Investment and Social Capital Participation: the former lays groundwork during early institutional reform through special budgets and bed capacity expansion, while the latter magnifies investment efficiency in mature phases with high reimbursement rates and private engagement. P3 underscores the critical role of Smart Service Platform Construction, indicating that under intense aging pressure, traditional supply side expansion alone is insufficient to meet diverse care needs and must be supplemented by digital services such as remote monitoring and AI assisted diagnosis. P4 illustrates the substitutive function of market mechanisms and insurance capital where fiscal support is limited, diversified capital and technological platforms can still sustain a high investment ratio.

Second, differences in policy pacing and institutional frameworks between Japan and South Korea explain coverage variations. South Korea’s higher coverage in P1, P2, and P4 reflects its more aggressive market reforms and insurance capital liberalization; Japan’s lead in the P3 Smart Service Platform Construction pathway stems from continuous investment in telecare pilots and AI technology demonstrations since 2017. Accordingly, countries or regions should dynamically adjust policy mixes: areas with robust fiscal capacity but weak market activity should prioritize incentives for social capital and technological innovation; fiscally constrained regions with solid private provision should ease capital entry and strengthen public private partnerships; and amid deep aging, Smart Service Platform Construction ought to be elevated in policy priority to enhance resource utilization efficiency through digital means.

Finally, despite advantages in data completeness and methodological innovation, this study has limitations. The panel dataset is confined to Japan and South Korea, and its external validity warrants testing through broader comparative research. Moreover, fsQCA excels at revealing causal path combinations but does not quantify the elasticity or marginal effects of individual factors. Future work could integrate in depth case interviews and randomized controlled trials to refine understanding of Smart Service Platform Construction and Social Capital Participation mechanisms, and extend analysis to provinces or additional countries to improve generalizability and policy operability.

### 5.2 Theoretical implication

Utilizing dynamic fsQCA, this research highlights the interactive and composite effects of five elements fiscal investment, social capital, technological innovation, digital platforms and institutional safeguards. Contrary to traditional single factor analyses, it finds that a high investment ratio is not driven by any solitary factor but by their mutual supplementation across multiple pathways. This conclusion resonates with the “synergy” concept in complexity governance theory, suggesting that scholars and policymakers should eschew isolated, tool based interventions in favor of a holistic, multi dimensional linkage framework that concurrently addresses fiscal levers, market mechanisms, technological support and social protection.

The results also demonstrate the prevalence of “equifinality” in eldercare investment: whether through government leadership plus capacity expansion, market driven high end technology, or digital empowerment with insurance capital engagement, each combination can yield high investment levels within its respective institutional environment. This aligns with integrated governance theory’s emphasis on accepting diverse pathways, underscoring the context dependency of policy outcomes and the need for evaluative models that categorize scenarios by institutional arrangements, demographic structures and fiscal capacity thereby improving replicability and adaptability of policy measures.

By comparing Japan and South Korea’s configurational coverage and pathway differences, the study validates the profound influence of institutional embeddedness and path dependence in public service reform. South Korea’s rapid market reforms and insurance capital openness, alongside Japan’s gradual evolution of smart platform pilots, both illustrate how institutional inertia and technological experimental fields shape policy development. Future theoretical research should integrate institutional embeddedness and technological innovation into dynamic evolution models, examine mechanisms for adjusting factor weights across development stages, and explore policy feedback pathways within multi level governance networks, thus constructing a more comprehensive theoretical framework for senior health public service reform.

### 5.3 Limitation and future research

Although based on annual panel data from 2014 to 2023 covering key supply demand variables in Japan and South Korea’s senior health sectors, this study has limitations. First, the annual frequency may obscure policy adjustments and short term fluctuations at quarterly or monthly intervals, potentially masking the effects of time sensitive innovation pilots. The seven condition variables exclude micro level factors such as family support and community mutual assistance, slightly reducing the model’s capacity to identify impacts of individual and social network behaviors. Dynamic fsQCA highlights configurational diversity and parallel pathways, yet regional heterogeneity in fiscal policy, demographic structure and digital development within each country is not fully captured at the national level, limiting the precision of cross regional policy recommendations.

To deepen understanding of diverse eldercare investment pathways, future research could: (1) incorporate quarterly or monthly data to capture dynamic policy implementation rhythms and short term shocks; (2) extend the analysis to other high and middle income countries to test external validity and institutional fit of configurational pathways; (3) include micro level factors such as family support, community mutual assistance and individual health behaviors to enrich the demand side perspective; (4) combine cost effectiveness and quality feedback indicators in mixed methods research to explore the trade off between resource allocation efficiency and service quality across different configurations; and (5) focus on institutional adjustments and long term effects following public health crises (e.g., the COVID 19 pandemic) to provide empirical support for building more resilient eldercare systems.

## Data Availability

Publicly available datasets were analyzed in this study. This data can be found at: https://www.korea.go.kr/ https://www.kantei.go.jp.
