# Supplementary figures and images for: Strategic investment patterns of the medical industry in senior health
Source: Front Public Health. 2025 Jul 11;13:1629981. doi: 10.3389/fpubh.2025.1629981 (PMC12289697; doi:10.3389/fpubh.2025.1629981)

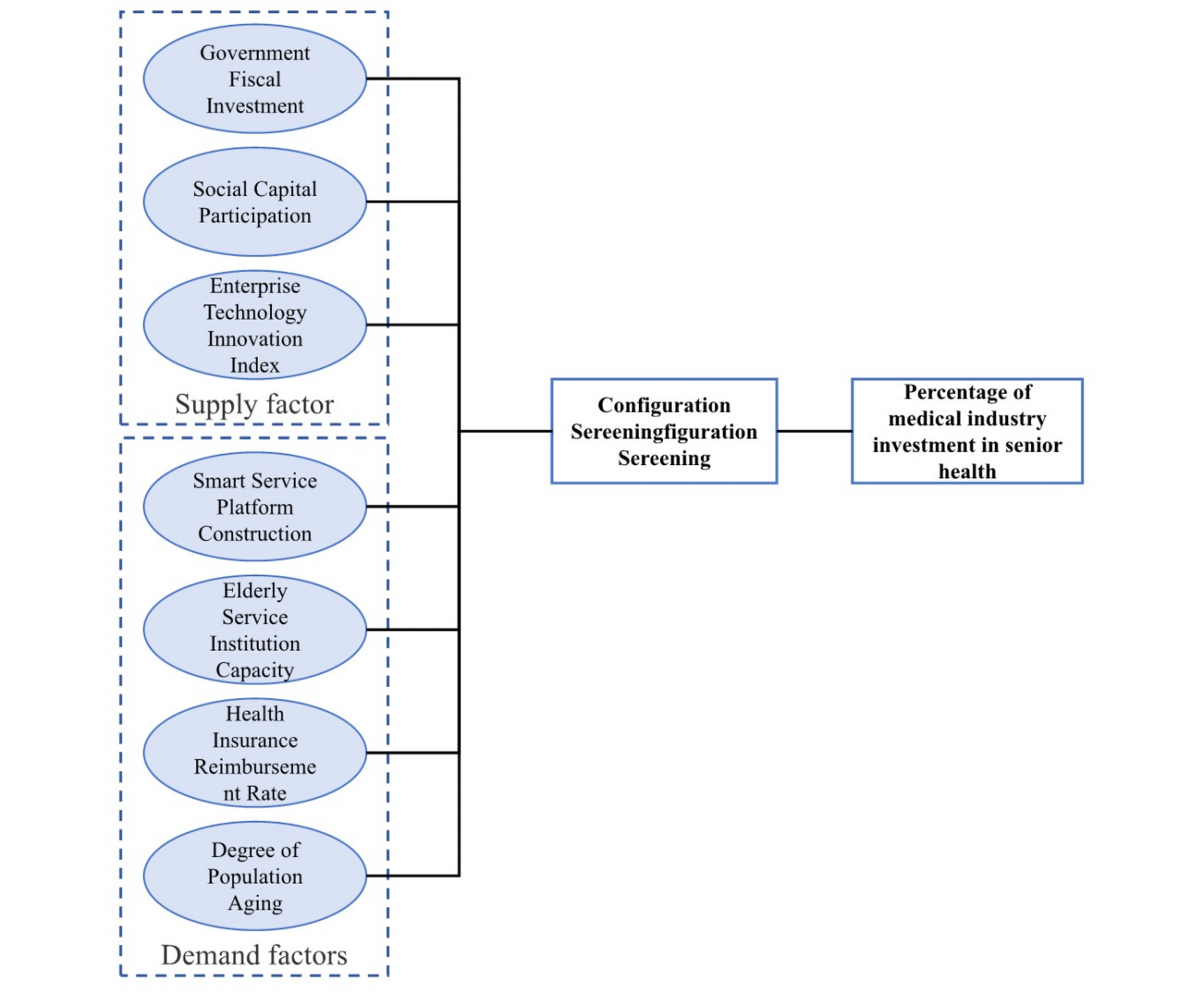

Supplement: Supplementary file 1 [file Image_1.PNG]
